# Impairment of GABA inhibition in insomnia disorders: Evidence from the peripheral blood system

**DOI:** 10.3389/fpsyt.2023.1134434

**Published:** 2023-02-09

**Authors:** Ting Xiang, Jiwu Liao, Yixian Cai, Mei Fan, Congrui Li, Xiaotao Zhang, Hongyao Li, Yushan Chen, Jiyang Pan

**Affiliations:** ^1^Sleep Medical Center, The First Affiliated Hospital of Jinan University, Guangzhou, Guangdong Province, China; ^2^Department of Psychiatry, The First Affiliated Hospital of Jinan University, Guangzhou, Guangdong Province, China

**Keywords:** insomnia, GABA, serum, GABA_A_ receptor, subunits

## Abstract

**Aim:**

To explore the change characteristics and related factors of various indexes of GABAergic system in peripheral blood of patients with insomnia disorder.

**Methods:**

In this study, a total of 30 patients who met the DSM-5 diagnostic criteria for insomnia disorder and 30 normal controls were included. All subjects had a structured clinical interview with the Brief International Neuropsychiatric Disorder Interview, and PSQI was used to evaluate the sleep status of the subjects. Enzyme-linked immunosorbent assay (ELISA) was used to detect serum γ-aminobutyric acid (GABA), and RT-PCR was used to detect GABA_A_ receptor α1 and α2 subunit mRNA. All data were statistically analyzed using SPSS 23.0.

**Results:**

Compared with the normal control group, the mRNA levels of GABA_A_ receptor α1 and α2 subunits in the insomnia disorder group were significantly lower, but there was no significant difference in the serum GABA levels between the two groups. And in the insomnia disorder group, there was no significant correlation between the GABA levels and the mRNA expression levels of α1 and α2 subunits of GABA_A_ receptors. Although no significant correlation was found between PSQI and serum levels of these two subunit mRNAs, its component factors sleep quality and sleep time were negatively correlated with GABA_A_ receptor α1 subunit mRNA levels, and daytime function was inversely correlated with GABA_A_ receptor α2 subunit mRNA levels.

**Conclusion:**

The inhibitory function of serum GABA in patients with insomnia may be impaired, and the decreased expression levels of GABA_A_ receptor α1 and α2 subunit mRNA may become a reliable indicator of insomnia disorder.

## Introduction

Insomnia disorder (ID) is a common sleep disorder, which means that patients are dissatisfied with the quality and/or amount of sleep and their daytime function is affected, although they have appropriate sleep environment and opportunities. The prevalence of insomnia disorder in European and American countries ranges from 6.9 to 27.3% (1–5), the prevalence of insomnia disorder among Chinese adults is about 15% in China (6). During the COVID-19 pandemic, the global prevalence of insomnia disorder reached 23.87% (7), and 29.2% in China (8). Insomnia disorder not only affects the social and cognitive functions of patients (9), but also increases the risk of mental diseases such as anxiety and depression, and physical diseases such as cardiovascular and cerebrovascular diseases, metabolic syndrome, and immune diseases (10). Additionally, sick leave, accidental injuries, and accidents caused by insomnia have brought heavy economic and medical burdens to patients, their families and society (11, 12).

γ-aminobutyric acid (GABA) is the main inhibitory neurotransmitter in the mammalian central nervous system, and no less than 20% of neurons are GABAergic neurons (13, 14). Under the action of glutamic acid dehydrogenase (GAD), glutamate (Glu) is decarboxylated to GABA. The maintenance of human normal sleep and wakefulness depends on the dynamic balance of the ascending activation system and descending inhibition system of the brainstem reticular structure, and GABA is the main material basis for maintaining this dynamic balance (15). GABA in the preoptic hypothalamus, especially in the ventrolateral preoptic area, can promote sleep, and GABA can also directly or indirectly inhibit arousal to maintain sleep (16, 17). In addition, agonists (benzodiazepines, non-benzodiazepines, etc.) that act on the α1, α2, α3, or α5 subunits of GABA_A_ receptors are commonly used clinically as drugs for the treatment of insomnia (15), which means GABAergic system may be involved in the development of insomnia. At present, studies on the correlation between GABAergic system and insomnia disorder mainly focus on the central nervous system, and few studies have explored the relationship between GABAergic system in peripheral blood system and insomnia disorder. Through Magnetic Resonance Spectroscopy (MRS) research, it was found that the overall level of GABA in the brain regions of patients with insomnia was lower than that of normal controls (18–20). Moreover, the research of Winkelman JW et al. showed that GABA levels were negatively correlated with Wake-time After Sleep Onset (WASO) (19). Although the study by Morgan et al. found that occipital GABA increased in insomnia disorder patients compared with normal controls, GABA remained negatively correlated with WASO. Therefore, they believed that the elevated occipital GABA levels in insomnia disorder patients may reflect an allogeneic response to chronic hyperarousal (21). Furthermore, some researchers improved the sleep quality of patients with insomnia disorder by increasing the content of peripheral blood GABA (22–25). However, we know little about the changes of peripheral blood GABAergic system in patients with untreated insomnia disorder. Therefore, in this study, we explored the peripheral blood serum GABA levels and the mRNA expression levels of GABA_A_ receptor α1 and α2 subunits in patients with insomnia disorders.

## Materials and methods

### Subjects

The patients with insomnia disorder came from outpatients and inpatients who visited the Department of Psychiatry of the First Affiliated Hospital of Jinan University from May 2018 to March 2019. Inclusion criteria: (1) Meet the diagnostic criteria of DSM-5 for insomnia disorder; (2) Age 18–65. (3) Pittsburgh Sleep Quality Index (PSQI) ≥ 8 points. (4) Junior high school and above. Exclusion criteria: (1) Patients combined with other sleep–wake disorders. (2) Patients with other mental disorders in the past and present. (3) Patients with brain organic diseases and other physical diseases. (4) Using antidepressants, antipsychotic drugs, and sleep-promoting drugs. (6) Users of long-acting antipsychotic drugs in the past 1 month. (7) Pregnant and lactating women.

The controls were healthy volunteers recruited from the community during the same period. Inclusion criteria: (1) The Mini-International Neuropsychiatric Interview Chinese version 5.0.0 (M.I.N.I.) structured interview did not meet the diagnostic criteria of DSM-5 for insomnia disorder. (2) Age between 18 and 65 years old. (3) PSQI≤7 points. (4) Junior high school and above. Exclusion criteria: (1) Those who combined with other sleep–wake disorders. (2) Those who with any mental disorders in the past and present. (3) Those who with brain organic diseases and other physical diseases. (4) Using antidepressants, antipsychotic drugs, and sleep-promoting drugs. (6) Users of long-acting antipsychotic drugs in the past 1 month. (7) Pregnant and lactating women. (8) Those who have blood relationship with the case group.

This study was reviewed and approved by the Ethics Committee of the First Affiliated Hospital of Jinan University, and all participants gave informed consent to this study and signed an informed consent form.

### Demographic information

The demographic data of all subjects were collected, and the occurrence and development of diseases in insomnia subjects were collected. The former includes gender, age, marital status, work status, family history of mental illness, etc. The latter includes the age of first onset, total disease duration, and current disease duration.

### Scale evaluation and diagnosis

All subjects were diagnosed consistent with the M.I.N.I. structured interview by two psychiatrists with intermediate professional titles, and then blood routine, liver function, kidney function, thyroid function, myocardial enzymes, electrocardiogram, abdominal B-ultrasound, head MR, and other related examinations were included in the study after excluding organic brain diseases and other physical diseases.

### Pittsburgh sleep quality index

It is used to evaluate the subjective sleep quality of the subjects in the last 1 month. Consists of 19 self-assessment items and five other-evaluation items, of which the 19th self-evaluation item and five other-evaluation items do not participate in scoring, 18 items form seven factors, and each factor is scored on a scale of 0–3, the cumulative score of each component is the total score of PSQI, the total score ranges from 0 to 2 l, and the higher the score, the worse the sleep quality. Seven factors were sleep quality, sleep latency, sleep time, sleep efficiency, sleep disturbance factors, use of hypnotic drugs, and daytime dysfunction (4).

### Sample collection and testing

All subjects collected 2 ml of fasting venous blood from the left elbow in dry tubes and EDTA anticoagulant tubes before receiving medication and physical therapy at 8–9 am on the next day after enrollment. The blood samples in the drying tube were left standing at 4°C for 30 min, centrifuged at 3,000 r/min at low temperature (4°C) for 10 min, and the supernatant was transferred to a sterile cryopreservation tube and stored in a −80°C refrigerator. The concentration of GABA was detected by enzyme-linked immunosorbent assay (ELISA), and the ELISA kit was provided by Guangzhou Blue Dolphin Biotechnology Co., Ltd. The concentration of the standard substance of the GABA kit was as follows: 8, 4, 2, 1, 0.5, and 0 μmol/L. Detection range 0.25–8 μmol/L Sensitivity: The lowest detection concentration was less than 0.1 μmol/L, the intra-assay coefficient of variation is 6%, and the inter-assay coefficient of variation is 11%.

Blood samples in EDTA anticoagulant tubes were used to detect the expression levels of GABA_A_ receptor α1 and α2 subunit mRNAs, and total RNA was extracted using the blood sample RNA extraction kit produced by OMEGA Company. At the same time, (1) purity test was completed: 1 μl RNA sample was diluted 50 times, and the OD value was measured on the BioPhotometer plus Eppendorf Nucleic Acid Protein Analyzer. The ratio of OD260/OD280 was greater than 1.8, indicating that the prepared RNA was relatively pure and free of protein contamination. (2) Integrity detection of total RNA: take 1 μl of RNA sample, electrophoresis on 1% agarose gel at 80 V × 20 min, observe the 5, 18, and 28 s rRNA bands of the total RNA with a gel imaging system, if the three bands are complete, it can be proved that the extraction of total RNA is relatively complete. cDNA was reverse-transcribed using a reverse transcription kit (provided by Guangzhou Blue Dolphin Biotechnology Co., Ltd.). According to the NCBI database sequence, the primers were synthesized by Shanghai Biochemical, and the primer sequences of the α1 and α2 subunits of the GABA_A_ receptor were designed as follows: α1 forward primer: 5′-GTCAAGCCCGAAACAAAACC, α1 reverse primer: GATTCCAAATAGCAGCGGGA-3′, α2 forward primer: 5′-TCGACATAGTCGTTGAAGCA, α2 reverse primer: GCAGGCACCCAAGATTAACA-3′. ABIPRISM®7500 sequence detection system was used to perform Reverse Transcription-PCR (RT-PCR) to measure mRNA levels. The RT-PCR reaction system included 5.0 μl of cDNA (1:20), 0.5 μl of upstream primers, 0.5 μl of downstream primers, 2xSYBR Green qPCRSuperMix (Invitrogen) 10 μl, dH2O 4.0 μl, 95°C for 5 min; 95°C for 15 s, 60°C for 32 s, and 40 cycles; melting curve analysis was performed after the cycle, and each sample was repeated three times. mRNA levels were calculated using the (2^−ΔΔCt^) method.

### Analysis

SPSS 23.0 was used for statistical analysis, and the statistics of measurement data were described as mean ± standard deviation and median (Lower quartile, Upper quartile). Count data were analyzed using chi-square test. Normally distributed measurement data, two groups were compared using *t*-test, non-normally distributed measurement data using *t*’ test. Correlation analysis between two variables was performed by Pearson correlation analysis or Spearman correlation analysis. All tests were two-sided, with a test level of α = 0.05.

## Results

### Demographic and clinical information

The results were shown in Table 1. This study included 30 patients in the insomnia disorder (ID) group, including 13 males. The age of ID group ranged from 18 to 65 years, with an average age of 39.13 ± 11.97. The total course of disease was 3 ~ 245 months, with a median of 25.00 (12.75, 79.50) months. The average age of first onset was 35.10 ± 9.69 years old. The duration of this onset was 2–93 months, with a median of 12.50 (4.00, 25.25) months. There were 30 subjects in the normal control (NC) group, 13 subjects were male. The age of NC group ranged from 20 to 64 years, with an average age of 34.67 ± 13.68 years. There was no significant difference in gender (*χ*^2^=0.000, *p* = 1.000) and age (*F* = 1.951, *p* = 0.183) between the two groups. There was a significant difference in marital status between the two groups (*F* = 8.864, *p* = 0.003).

**Table 1:** Demographic characteristics of subjects.

Characteristics	ID	NC	*p* values
Number of subjects	30	30	-
Age (years) Mean ± SD	39.13 ± 11.97	34.67 ± 13.68	0.183
Age range (years)	18–65	20–64	-
Gender (male/female)	13/17	13/17	1.000
Married (yes/no)	25/5	14/16	0.003
Family history (yes/no)	3/27	0/30	0.236
First-episode (yes/no)	21/9	-	-
Total duration of illness (month)	25.00 (12.75–79.50)	-	-
Age of first onset	35.10 ± 9.69	-	-
Time of this course (month)	12.50 (4.00–25.25)	-	-

ID, insomnia disorder group; NC, normal control group; SD, standard deviation.

### Comparison of GABA levels and the mRNA levels of GABA_A_ receptor α1 and α2 subunits between insomnia disorder group and normal control group

**Figure 1:**
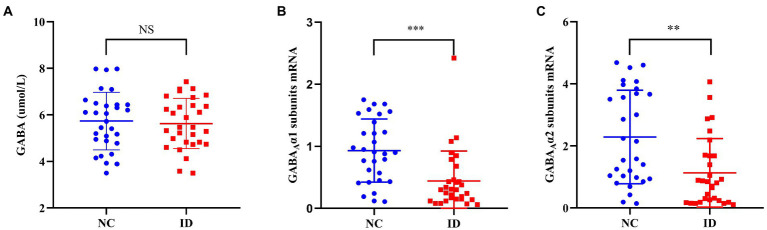
Comparison of γ-aminobutyric acid (GABA) levels and the mRNA levels of GABA_A_ receptor α1 and α2 subunits between ID and NC. NS, no significant; ID, insomnia disorder group; NC, normal control group. ^**^p < 0.01. ^***^p < 0.001.

There was no significant difference in serum GABA levels between ID group and NC group (*F* = 0.458, *p* = 0.733). However, compared with the NC group, the peripheral blood GABA_A_ receptor α1 (*F* = 1.573, *p* < 0.001) and α2 subunits (*F* = 8.757, *p* = 0.001) levels in the ID group were significantly decreased (Figure 1).

### Analysis of related factors of serum the serum mRNA levels of GABA_A_ receptor α1 and α2 subunits in the insomnia disorder group

As shown in Table 2, we found that the mRNA expression levels of GABA_A_ receptor α1 and α2 subunits were positively correlated with the age (*r* = 0.462, *p* < 0.05; *r* = 0.483, *p* < 0.01) and the age of first onset (*r* = 0.498, *p* < 0.01; *r* = 0.454, *p* < 0.05) of insomnia disorder patients, but no significant correlation was found between these two indicators and gender, total disease duration, and PSQI total score. We also analyzed the relationship between the expression levels of GABA_A_ receptor α1 and α2 subunits mRNA and PSQI factors, and found that the expression level of GABA_A_ receptor α1 subunits mRNA was negatively correlated with sleep quality (*r* = −0.383, *p* < 0.05) and sleep time (*r* = −0.381, *p* < 0.05), while there was a negative correlation between the expression of GABA_A_ receptor α2 subunits mRNA and daytime function (*r* = −0.491, *p* < 0.01).

**Table 2:** Analysis of related factors of serum the serum mRNA levels of GABA_A_ receptor α1 and α2 subunits in the insomnia disorder group.

Variable	GABA_A_ α1 subunits mRNA		GABA_A_ α2 subunits mRNA	
	*r*	*p*	*r*	*p*
Age	0.462^*^	0.010	0.483^**^	0.007
Gender	0.121	0.524	0.244	0.193
Total duration of illness	0.150	0.163	0.307	0.099
Age of first onset	0.498^**^	0.005	0.454^*^	0.012
PSQI	−0.200	0.290	−0.070	0.715
Sleep quality	−0.383^*^	0.037	−0.325	0.080
Sleep latency	0.078	0.683	0.190	0.314
Sleep time	−0.381^*^	0.038	−0.147	0.440
Sleep efficiency	−0.219	0.244	−0.008	0.967
Sleep disturbance factor	0.126	0.509	0.343	0.064
Drugs	0.178	0.346	0.070	0.712
Daytime function	−0.269	0.150	−0.491^**^	0.006

^*^p < 0.05; ^**^p < 0.01.

### Correlation analysis of serum GABA levels, GABA_A_ receptor α1 subunits mRNA and GABA_A_ receptor α2 subunits mRNA expression levels in the insomnia disorder group

**Figure 2:**
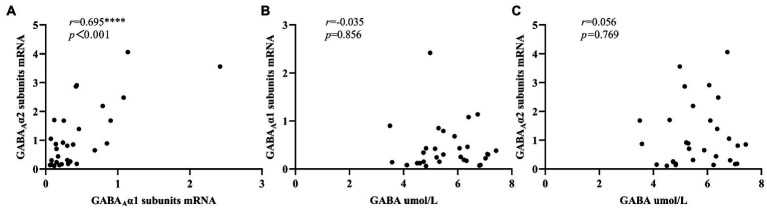
Correlation analysis of serum GABA level, GABA_A_ receptor α1 subunits mRNA and GABA_A_ receptor α2 subunits mRNA expression levels in the insomnia disorder group. 2^–∆∆Ct^ was used to calculate the relative expression of GABA_A_ receptor α1 subunits mRNAs and GABA_A_ receptor α2 subunits mRNAs. ^****^p < 0.0001.

**Figure 3:**
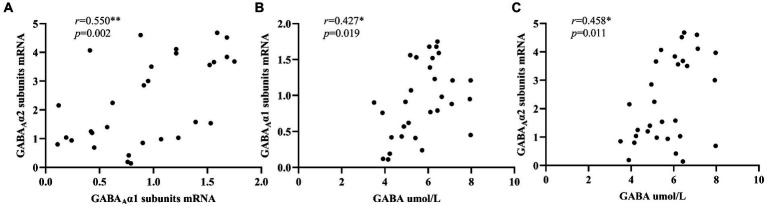
Correlation analysis of serum GABA level, GABA_A_ receptor α1 subunits mRNA and GABA_A_ receptor α2 subunits mRNA expression levels in the normal control group. 2^–∆∆Ct^ was used to calculate the relative expression of GABA_A_ receptor α1 subunits mRNAs and GABA_A_ receptor α2 subunits mRNAs. ^*^p < 0.05. ^**^p < 0.01.

Figure 2A showed that there was a significant positive correlation between the expression levels of peripheral blood GABA_A_ receptor α1 subunits mRNA and GABA_A_ receptor α2 subunits mRNA in ID group (*r* = 0.695, *p* < 0.001), while no significant correlation was found between serum GABA levels and the mRNA expression levels of these two subunits (Figures 2B,C). In addition, in the NC group (Figure 3), we found statistically significant correlations between peripheral blood GABA_A_ receptor α1 subunits mRNA and GABA_A_ receptor α2 subunits mRNA expression, between the serum GABA levels and GABA_A_ receptor α1 subunits mRNA expression, between the serum GABA levels and the mRNA expression levels of GABA_A_ receptor α2 subunits.

## Discussion

Our study did not find significant differences in serum GABA levels between insomnia disorder group and normal control group, but there were significant differences in the expression levels of peripheral blood GABA_A_ receptor α1 subunits mRNA and GABA_A_ receptor α2 subunits mRNA between the two groups. Moreover, in the ID group, the serum GABA levels did not seem to be significantly correlated with the expression of GABA_A_ receptor α1 subunits mRNA and GABA_A_ receptor α2 subunits mRNA, but in the NC group, there was a positive correlation between the serum GABA levels and the expression of these two subunits. In addition, the expression levels of GABA_A_ receptor α1 subunits mRNA and GABA_A_ receptor α2 subunits mRNA in peripheral blood were significantly positively correlated between the two groups, and the ID group was more significant.

The GABA_A_ receptor is composed of five subunits and belongs to the ligand-gated ion channel family, which is activated after binding to the inhibitory neurotransmitter GABA to play a sleep-promoting role (26). The three generations of hypnotics used clinically are all based on the inhibition process mediated by GABA_A_ receptors (27). There are 19 subunit types of GABA_A_ receptors (α1-6, β1-3, γ1-3, δ, ε, θ, π, and ρ1-3) (28), and many GABA_A_ receptors consist of two α subunits, two β subunits, and one γ subunit, α1β2γ2 type accounts for about 60% of GABA_A_ receptors, followed by α2β3γ2 type accounting for 15–20% (29, 30). The benzodiazepine binding site is formed by one of the α1, α2, α3, and α5 subunits with the γ subunit, while non-benzodiazepines preferentially bind to α1βγ2-type GABA_A_ receptor (31). Although the current clinically preferred non-benzodiazepines are known to bind preferentially to the α1-GABA_A_ receptor, they can still bind to the α2-GABA_A_ receptor and the α3-GABA_A_ receptor. The study by Crestani et al. suggested that the sedative-hypnotic and anticonvulsant activity of zolpidem was due to its action on α1-GABA_A_ receptors rather than α2- or α3-GABA_A_ receptors (32). However, the study by Kopp et al. showed the opposite. Their results showed that the non-benzodiazepine drug-zolpidem seems to produce sedative-hypnotic effects after binding to α2-GABA_A_ receptor and/or α3-GABAA receptor, but not to α1-GABAA receptor combination produced (33). In addition, Uygun et al. also suggested that the ability of zolpidem to reduce NREM sleep latency and increase sleep time may be related to α2-GABA_A_ receptors (34). This means that GABA_A_ receptors, especially α1-GABA_A_ and/or α2-GABA_A_ receptor may play an important role in the pathophysiological process of sleep, and the disturbance of GABAergic system may cause insomnia. Our study suggested that compared with the NC group, the expression levels of peripheral blood GABA_A_ receptor α1 subunits mRNA and GABA_A_ receptor α2 subunits mRNA in the ID group were significantly decreased, and GABA_A_ receptor was activated to participate in the occurrence of sleep, and their expression decreased, which meant sleep drive and maintenance were disrupted, resulting in insomnia. α1-GABA_A_ receptor and α2-GABA_A_ receptor in peripheral blood may be used as biomarkers of insomnia, of course, it still needs a large number of samples to verify.

Our study also found that compared with the NC group, the GABA levels of the ID group did not observe significant changes, but the receptor expression of the latter decreased, which meant that although the GABA content in the serum of the patients remained unchanged, the number of receptors that can interact with GABA decreases, and the inhibitory effect of GABA was also affected. The expression levels of GABA_A_ receptor α1 subunits mRNA and GABA_A_ receptor α2 subunits mRNA in the ID group had no significant relationship with GABA, but there was a positive correlation in the NC group, which suggested that the GABAergic system of normal individuals had homeostatic self-regulation, while the peripheral blood GABA system of the ID group was damaged and could not regulate the balance of GABA levels and its receptors. Since GABA hardly crosses the blood–brain barrier (35, 36), serum GABA levels do not directly reflect GABA levels in the central nervous system. Then it could also explain the inconsistency between our results and the reduction of GABA levels in the central nervous system of patients with insomnia (18–20). However, some studies have found that some herbal medicine extracts can shorten the sleep latency and maintain sleep by increasing the level of GABA and the expression level of GABA_A_ receptor α1 protein in mouse serum and brain tissues (37). Moreover, the combined intake of GABA and L-theanine increased the level of GABA in rat brain tissues and increased the expression of GABA_A_ receptor, thereby promoting sleep and reversing the sleep reduction caused by caffeine in rats (38). This mechanism of improving sleep by increasing peripheral blood GABA levels may be produced through indirect pathways, such as through the enteric nervous system (ENS) (39) or the possible presence of GABA transporters in the blood–brain barrier (40).

Through correlation analysis, we found that the age of the ID group and the age of first onset were positively correlated with the expression of GABA_A_ receptor α1 and α2 subunits mRNA. In addition, we did not find a significant correlation between the age of the NC group and the expression of these two subunits (*r* = 0.268, *p* = 0.152; *r* = 0.219, *p* = 0.245), which indicated that age might affect the expression of GABA_A_ receptor α1 and α2 subunits mRNA in some way in the insomnia disorder patients, but there was no relevant research report so far. At the same time, our results also suggested that the patient’s subjective sleep quality assessment total score (PSQI) seemed to have no significant correlation with the peripheral blood expression of these two subunits. However, by analyzing the correlation between the PSQI factor scores and the expression levels of GABA_A_ receptor α1 and α2 subunits mRNA, we found that the worse the sleep quality and the less sleep time, the lower the serum expression of α1-GABA_A_ receptor. The worse the daytime function, the lower the serum level of GABA_A_ receptor α2 subunits mRNA. Agosto et al. confirmed that GABA_A_ receptor promotes the initiation of sleep (41), so we inferred that the activation of GABA_A_ receptor can prolong the total sleep time by reducing the sleep latency and improve the patient’s subjective sleep satisfaction. Moreover, our results were consistent with Crestani et al.’s suggestion that activation of α1-GABA_A_ receptors may mediate sedative-hypnotic and anticonvulsant (32). Some studies have found that the expression imbalance of GABA_A_ receptor α subunit may be related to cognitive function (42), and GABA_A_ receptor blockade can impair social behavior and attention, but the specific subunit type needs to be further determined (43).

Our study also has certain limitations. We only measured the levels of peripheral serum GABA and GABA_A_ receptor α1 and α2 subunits mRNA, and only used subjective assessment scales to evaluate the sleep and function of patients. In the future, while increasing the number of samples to verify the above results, we also need to measure the levels of the above indicators in the central nervous system, and combine objective evidence to explore the correlation between patients’ insomnia symptoms and severity and the GABA system.

## Conclusion

In summary, our research showed that the inhibition of GABA in the peripheral blood system of patients with insomnia disorder was impaired, which might be mediated by the abnormal expression of its receptor subunits. In future research, we can focus on the function of receptors in the peripheral blood system to explore the clinical characteristics of insomnia disorder.

## Data availability statement

The raw data supporting the conclusions of this article will be made available by the authors, without undue reservation.

## Ethics statement

The studies involving human participants were reviewed and approved by IRB of the First Affiliated Hospital of Jinan University. The patients/participants provided their written informed consent to participate in this study.

## Author contributions

TX was responsible for research concept and design, literature research, manuscript preparation, and data analysis. JL was responsible for research concept and design, literature research, and experimental research. YiC was responsible for statistical analysis, mapping, and data collection. MF and CL were responsible for clinical research. XZ, HL, and YuC were responsible for the sleep assessment of subjects. JP was responsible for the integrity and manuscript review of the entire study. All authors contributed to the article and approved the submitted version.

## Funding

This research was supported by National Natural Science Foundation of China (grant number: 81871036).

## Conflict of interest

The authors declare that the research was conducted in the absence of any commercial or financial relationships that could be construed as a potential conflict of interest.

## Publisher’s note

All claims expressed in this article are solely those of the authors and do not necessarily represent those of their affiliated organizations, or those of the publisher, the editors and the reviewers. Any product that may be evaluated in this article, or claim that may be made by its manufacturer, is not guaranteed or endorsed by the publisher.
